# Implementing intranasal povidone-iodine in the orthopedic trauma surgery setting to prevent surgical site infections: a qualitative study of healthcare provider perspectives

**DOI:** 10.1186/s13756-025-01526-5

**Published:** 2025-02-21

**Authors:** AM Racila, Erin C. Balkenende, Loreen A. Herwaldt, Michael C. Willey, Linda D. Boyken, Jean G. Pottinger, Brennan S. Ayres, Kimberly C. Dukes, Melissa A. Ward, Marin L. Schweizer

**Affiliations:** 1 Department of Internal Medicine, University of Iowa Roy J and Lucille A Carver College of Medicine, Iowa City, IA USA; 2 Center for Access & Delivery Research and Evaluation (CADRE), Iowa City VA Health Care System, Iowa City, USA; 3 Division of Infectious Disease, University of Wisconsin, Madison, USA

## Abstract

**Background:**

Surgical site infections (SSIs) are associated with morbidity, mortality, and increased costs. *Staphylococcus aureus* is the most common cause of SSIs and approximately 30% of hemodialysis patients carry this organism in their nares. Unlike mupirocin, intranasal povidone-iodine (PVI) is applied only the day of surgery to prevent surgical site infections. Thus, intranasal PVI could be valuable in orthopedic trauma surgery settings where time to prepare a patient for surgery is limited.

**Methods:**

We conducted a small phase IV post-marketing study from 2020 to 2021 in an academically affiliated hospital wherein staff administered intranasal PVI pre- and post-operatively to consenting patients undergoing orthopedic fixation procedures for traumatic fractures. Before implementing the PVI intervention, we conducted a human factors task analysis to determine the optimal time and hospital location to perform PVI decolonization for patients receiving these orthopedic fixation procedures. After the post-marketing study was completed, we conducted qualitative interviews with healthcare staff to determine barriers and facilitators that could affect staff members’ likelihood of administering PVI to patients. We aligned our inductive interview findings with strategies defined in Powell and colleagues’ Expert Recommendations for Implementing Change (ERIC) framework to facilitate generalizability and standardized reporting of implementation strategies.

**Results:**

Our human factors task analysis identified the Day of Surgery Admissions (DOSA) as the appropriate context for PVI administration within surgical workflow, as there was downtime during this period and direct patient-provider communication could occur. Two DOSA nurses, one postoperative nurse, and one orthopedic trauma surgeon agreed to be interviewed. Facilitators of intranasal PVI administration included emphasizing the non-invasiveness of PVI nasal swabs to patients and emphasizing intranasal PVI efficacy to staff and patients. While the nurse participants felt that having PVI orders with other medication orders in the EMR helped them identify patients enrolled in the study and who required PVI, entering these orders increased the surgeon’s workflow and presented a time barrier.

**Conclusions:**

Macro- and micro-level contextual factors should be considered when tailoring implementation to healthcare settings. Our findings reinforce prior work demonstrating the value of incorporating human factors engineering methodologies into infection control and prevention implementation approaches.

## Background

Surgical site infections (SSIs) are associated with morbidity, mortality and increased costs [1]. *Staphylococcus aureus* is the most common cause of SSIs, and approximately 30% of patients carry this organism in their nares [2, 3]. Evidence-based strategies can prevent approximately half of all SSIs [4]. These strategies are often implemented as surgical care “bundles” that may include steps such as pre-operative skin preparation, healthcare worker double-gloving, or prophylactic antibiotics [5]. 

We previously implemented a surgical care bundle among patients having hip, knee, or cardiac surgery enrolled in a multicenter study that included screening for *S. aureus* nasal colonization, decolonizing carriers with intranasal mupirocin and chlorhexidine gluconate body wash, and prescribing optimal perioperative antibiotic prophylaxis [6]. The intervention decreased the rate of complex *S. aureus* SSIs 42% (rate ratio = 0.58; 95% CI, 0.37 to 0.92). However, compliance with intranasal mupirocin was not ideal. Mupirocin should be applied twice daily for five days, which was difficult to implement within the non-elective, expeditious context of orthopedic trauma surgery. Both mupirocin and povidone-iodine (PVI), when applied intranasally before surgery, reduce rates of SSI [2, 7]. Maslow et al. found that patients who received PVI reported fewer adverse events than those who received mupirocin for perioperative prophylaxis [8]. While use of mupirocin can lead to development of resistance, PVI has been used in healthcare settings for decades and resistance has not been found [9, 10]. In addition, PVI can be applied 1 h before a procedure, which makes it easier to use than mupirocin for perioperative decolonization [3]. Thus, PVI could be valuable in orthopedic trauma surgery settings, where time to prepare a patient for surgery is limited.

We conducted a small phase IV post-marketing study from 2020 to 2021 in an academically affiliated hospital wherein staff administered intranasal PVI (10% w/w PROFEND, PDI Healthcare, Woodcliff Lake, NJ) pre- and post-operatively to consenting patients undergoing orthopedic fixation procedures for traumatic fractures [11]. Patients in the study received nasal PVI in the hour before surgery, then in the evening after surgery. Before implementing the intervention, we conducted a human factors task analysis to determine the optimal time and hospital location at which to perform PVI decolonization for patients having orthopedic fixation procedures for traumatic fractures. After the post-marketing study was completed, we conducted qualitative interviews with staff to determine barriers and facilitators that could affect staff members’ likelihood of administering the PVI. We present the results of the task analysis and the interviews and discuss the pre- and post-surgical context, and compare these findings with facilitators identified during our human factors engineering task analysis.

## Methods

### Identifying the Appropriate Context for PVI Administration

We conducted a human factors task analysis to identify which staff roles (e.g., day of surgery nurse, operating room nurse) should apply PVI; where and at which time PVI should be applied; how to support adherence to PVI administration; and where PVI should be stored. Human factors engineer BSA observed practices and performed a timeline analysis, which involved timing the actions of all staff caring for the patients during this time period. BSA followed five patients: two patients from admission to Day of Surgery Admissions (DOSA) until the patients were brought to the operating room (OR) suite, and three patients from their admission to DOSA, transfer to OR, and up to the time of incision.

### Interview setting, timing, & recruitment

In 2021, we conducted semi-structured interviews with healthcare professionals (HCPs) who either administered PVI directly to patients or were otherwise involved in initiating PVI administration for our post-marketing study intervention. We contacted potential participants by e-mail and interested HCPs scheduled their interviews by phone. We used a purposive sampling approach as our goal was to elicit opinions about the PVI intervention across staff roles.

### Data collection & analysis

We recruited participants via e-mail and conducted all interviews by phone. Interviews ranged from 13 to 27 min and lasted on average 20 min. Interviews were audio-recorded with permission and transcribed. Our interview guide included questions about participants’ and patients’ perceptions of PVI administration, feasibility in the orthopedic trauma surgery setting, and suggestions for improving PVI implementation.

We recorded, transcribed, deidentified, and uploaded all transcripts into the qualitative software MAXQDA 2020 for analysis [12]. We coded responses via an inductive approach to categorize main themes. ECB and AMR initially independently coded the interview data and subsequently met periodically to corroborate emerging themes. We aligned our inductive findings with strategies defined in Powell et al.’s Expert Recommendations for Implementing Change (ERIC) implementation strategies framework to facilitate study generalizability and standardized reporting of implementation strategies [13]. 

### Ethics

This qualitative study was approved by the University of Iowa Institutional Review Board. We obtained verbal informed consent from all participants. Participants were eligible for a $30 gift card as compensation for completing interviews.

## Results

### Human factors task analysis

**Fig. 1:**
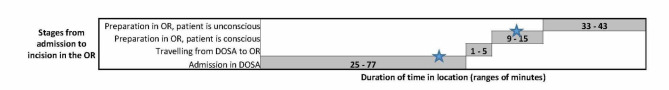
Timeline from the time patients entered DOSA until their incisions were made (*n* = 3). The blue stars represent possible points in the process when patients are conscious and PVI could be administered.

The workflow and time required for specific tasks or during specific time periods varied from case to case (Fig. 1). Workflow in DOSA involved 6–7 staff who sequentially did specific tasks. Workflow in the operating room while patients were conscious involved 8–9 staff who performed multiple tasks simultaneously. The sequential workflow was the main advantage of applying PVI in DOSA. However, if a case is delayed, the time from application to incision could be longer than optimal.

The time proximity to the incision and the circulating nurses’ interactions with conscious patients were advantages to applying PVI in the operating room; however, the complex workflow in the operating room could increase the likelihood that PVI would not be given. The task and timeline analyses suggested that either the DOSA nurse or the circulating nurse would be best situated to apply PVI depending on whether PVI is applied in DOSA or the operating room. In DOSA, the patient is conscious and there is more downtime. Given that the manufacturer recommends administering PVI within one hour before surgery, we chose to apply PVI near the end of the patient’s time in DOSA.

### Semi-structured interviews

We conducted semi-structured qualitative interviews with four healthcare workers. Two DOSA nurses, one postoperative nurse, and one orthopedic trauma surgeon agreed to be interviewed.

### Visual aids facilitated PVI administration

Two of the nurses indicated that visual aids facilitated PVI administration. A poster listing step-by-step instructions for PVI administration was available inside a medication room and this poster served as a “refresher” for a nurse who administrated PVI infrequently during the study. She stated, “…I don’t need like a rundown like every little tiny detail, I need like how many times do I do it and for how long, you know? I feel like that poster was very like succinct and short and it was like okay, I got it” (Postoperative Nurse). This nurse found the visual poster more useful than the text-based educational materials our researchers provided to staff at the beginning of the intervention. Another staff nurse participant suggested that a sign in the patient’s room describing the purpose of nasal PVI or a patient information pamphlet would encourage patients to accept the intranasal PVI.

### Low staff and patient burden facilitated PVI implementation into existing workflow

All three nurses agreed that while integrating PVI into their existing workflow added a few minutes of work, they believed staff would eventually adopt the intervention because the time required to apply the PVI is minimal, the swab is noninvasive, and intranasal PVI could prevent infections. Nurses already administer several pre-operative infection prevention precautions to patients, including chlorhexidine bathing for all surgical patients and routine MRSA and COVID nasal screening. Staff were willing to integrate PVI into their infection prevention practices due to its role in infection prevention. A DOSA nurse noted, “It just seems like part of the process you know; this is what we do to get you ready.”

### Having patients self-administer PVI would facilitate staff workflow

Patients sometimes confused the PVI and COVID swabs, and staff offered solutions for increasing the likelihood that patients would find having PVI applied to their nares acceptable. For example, one nurse noted that patients were more receptive to the PVI swab after she explained that the swab would not extend beyond the inside of the nares. A different staff nurse suggested that having patients self-administer the PVI swab would ease workflow and give patients more control over the process: “…instead of talking about why it would be uncomfortable, if [patients] just kind of did it themselves… I think it would be quicker accepted and easier for the patients” (DOSA Nurse).

### Emphasizing PVI efficacy to staff and patients incentivized adoption

When asked what could make the intervention more successful, all four interviewees stated that showing staff and patients evidence that use of PVI prevents SSI infection would facilitate PVI adoption. A DOSA nurse noted,

You know does it take a few more minutes? Yes. Um…but is it worth it? Probably. You know if the research shows that it’s worth it and it helps prevent infection then it’s worth doing.

The orthopedic surgeon also suggested that educating staff about the evidence for PVI’s efficacy would facilitate implementation in this hospital and in others. Similarly, the four participants indicated that educating patients about PVI’s efficacy would encourage patients to accept this intervention. A DOSA nurse remarked that, “I love the research part of it. So if a patient asked us questions we could look professional in answering that question, this is why we’re using it, we appreciate you participating in it and these are the outcomes that we are experiencing and explain to them in terms that they can understand the effectiveness…”.

### Including nasal PVI with medications in EMR facilitated implementation for some staff roles

The nurse participants felt that having PVI orders with other medication orders in the electronic medical record (EMR) was a key facilitator because it helped them identify the patients who were enrolled in the study and required PVI. A DOSA nurse described the process this way: “If the swabs were ordered pre-op, they would already be in the order set so that we knew that the patient was… a patient that was going to be in the study and we need to do the swabs so it kind of gave us the heads up that the patient would have that.” In contrast, the orthopedic trauma surgeon—who was tasked with entering PVI orders for each patient—found that it increased his workload because placing the order took 2 to 5 min per patient. The surgeon suggested that PVI be included as a standing order for these patients to reduce this implementation barrier.

### Barrier and facilitator concordance with the Expert Recommendations for Implementing Change (ERIC) Framework

Several of the barriers and facilitators emerging from our analysis were concordant with implementation strategies identified in Powell’s Expert Recommendations for Implementing Change (ERIC) framework [13]. Concordant implementation strategies included *conducting educational outreach visits; distributing educational material; involving patients/consumers and family members in the intervention; reminding clinicians; and promoting adaptability.* We list these corresponding strategies in Table 1 with examples from our orthopedic trauma surgery context.

**Table 1:** Powell and colleagues’ Expert Recommendations for Implementing Change (ERIC) framework strategies concordant with those emerging from PVI administration in the studied orthopedic trauma surgery setting

ERIC Strategy	Strategy Description	Relevance to PVI Administration in the Orthopedic Trauma Surgery Setting
Conducting educational outreach visits	Have a trained person meet with providers in their practice settings to educate providers about the clinical innovation with the intent of changing the provider’s practice.	*The research team facilitated the intervention by conducting outreach visits to the DOSA and the hospital units at the beginning of the study and modeling appropriate PVI administration procedures for staff.*
Distributing educational materials	Distribute educational materials (including guidelines, manuals, and toolkits) in person, by mail, and/or electronically.	*A poster hanging in the medication room*,* which showed how to apply PVI to patients’ nares*,* served as a quick reference for the nurses.* *Evidence-based justification for nasal PVI facilitated implementation.*
Involving patients/consumers and family members in the intervention	Engage or include patients/consumers and families in the implementation effort	*Interviewees suggested that patients would be less apprehensive about using PVI swabs in the COVID-19 context if patients had the option to self-administer the PVI.*
Remind clinicians	Develop reminder systems designed to help clinicians to recall information and/or prompt them to use the clinical innovation.	*The PVI order in the medication section of the EMR reminded nurses to administer the PVI.*
Promote adaptability	Identify the ways a clinical innovation can be tailored to meet local needs and clarify which elements of the innovation must be maintained to preserve fidelity.	*The preloaded PVI swabs facilitated easy*,* rapid*,* and non-invasive application in patients’ nares and*,* thereby*,* facilitated its integration into existing infection prevention protocols included in the nurses’ workflow.*

## Discussion

Our human factors task analysis and qualitative interviews with two DOSA nurses, one postoperative nurse, and one orthopedic surgeon clarified barriers and facilitators to implementing intranasal PVI to prevent SSI among patients undergoing surgical procedures for orthopedic trauma. Participants identified passive and active strategies that would facilitate PVI administration, including dissemination of educational materials to patients and providers, educational outreach visits, and reminders [14]. 

Our semi-structured interview findings substantiate the results from our human factors engineering task analysis, which identified DOSA as an appropriate context for PVI administration within the surgical workflow. Most of our identified facilitators could occur only while patients were conscious, including integrating PVI swabs into existing infection control bundles, assuring patients that PVI swabs touched only the nostrils not the nasopharynx like COVID-19 swabs, and communicating the efficacy of PVI to patients. These facilitators also required workflow downtime and direct patient-provider communication. Our findings reinforce previous work demonstrating the value of integrating human factors engineering methodologies into infection control and prevention implementation approaches [15, 16]. 

Checklists and similar tools that streamline preoperative workflow by prompting staff to act at important points in the surgical process have been shown to improve patient safety outcomes [17, 18]. A recent systematic review of implementation strategies to prevent SSIs after abdominal surgery found that manual and computerized interventions prompting HCPs to perform actions was one of five key strategies that meaningfully reduced SSI risk [19]. In the current study, facilitators included the instructional poster and the inclusion of nasal PVI in the EMR’s patient medication list, which reminded nursing staff to administer PVI to consenting patients after their operations. Although nurses liked the PVI orders, the orthopedic surgeon identified entering PVI orders into the EMR as an implementation barrier, which indicates that HCPs must examine contextual differences, including staff role and workflow, when developing infection prevention interventions [20]. 

HCPs in our study indicated that communicating the evidence supporting nasal PVI’s efficacy for reducing infection risk would improve PVI adoption, and thus would be a key implementation strategy. Interviewees stated that the information should be presented in an easily digestible way so HCPs could educate patients about PVI’s purpose and benefit.

## Conclusions

Macro- and microlevel contextual factors—such as shifts in perception of PVI swab invasiveness within the context of the COVID-19 pandemic and role-specific barriers and facilitators to PVI implementation—are key to consider when tailoring implementation efforts to individual healthcare settings [21, 22]. 

### Limitations

This study has limitations. Only four healthcare workers responded to email requests to participate in the interview. While we conducted the interviews in accordance with respondents’ schedules, interviews took place following the height of the COVID-19 pandemic and healthcare burnout and fatigue likely played a role in our low response rate. Voluntary response bias was also possible, as we may have interviewed HCPs who had strong opinions about implementing nasal PVI in their workplace. Additionally, some of our findings may not apply outside an orthopedic trauma surgical setting in a Midwestern academic hospital.

## Data Availability

In order to protect study participant data, the data in this study cannot be shared openly.
