# Correction: Implementing intranasal povidone-iodine in the orthopedic trauma surgery setting to prevent surgical site infections: a qualitative study of healthcare provider perspectives

**DOI:** 10.1186/s13756-025-01621-7

**Published:** 2025-08-14

**Authors:** A. M. Racila, Erin C. Balkenende, Loreen A. Herwaldt, Michael C. Willey, Linda D. Boyken, Jean G. Pottinger, Brennan S. Ayres, Kimberly C. Dukes, Melissa A. Ward, Marin L. Schweizer

**Affiliations:** 1 Department of Internal Medicine, University of Iowa Roy J and Lucille A Carver College of Medicine, Iowa City, IA USA; 2 Center for Access & Delivery Research and Evaluation (CADRE), Iowa City VA Health Care System, Iowa City, USA; 3 Division of Infectious Disease, University of Wisconsin, Madison, USA

Antimicrobial Resistance & Infection Control (2025) 14:14

10.1186/s13756-025-01526-5

The original article has been corrected to amend a typo in co-author, Brennan Ayres’s name.

